# A Novel 1,4-Dihydropyridine Derivative Improves Spatial Learning and Memory and Modifies Brain Protein Expression in Wild Type and Transgenic APP_SweDI_ Mice

**DOI:** 10.1371/journal.pone.0127686

**Published:** 2015-06-04

**Authors:** Baiba Jansone, Inga Kadish, Thomas van Groen, Ulrika Beitnere, Doyle Ray Moore, Aiva Plotniece, Karlis Pajuste, Vija Klusa

**Affiliations:** 1 Department of Pharmacology, Faculty of Medicine, University of Latvia, Riga, Latvia; 2 Department of Cell, Developmental and Integrative Biology, University of Alabama at Birmingham, Birmingham, Alabama, United States of America; 3 Department of Pharmacology/Toxicology, University of Alabama at Birmingham, Birmingham, Alabama, United States of America; 4 Latvian Institute of Organic Synthesis, Riga, Latvia; Hungarian Academy of Sciences, HUNGARY

## Abstract

Ca^2+^ blockers, particularly those capable of crossing the blood-brain barrier (BBB), have been suggested as a possible treatment or disease modifying agents for neurodegenerative disorders, e.g., Alzheimer’s disease. The present study investigated the effects of a novel 4-(N-dodecyl) pyridinium group-containing 1,4-dihydropyridine derivative (AP-12) on cognition and synaptic protein expression in the brain. Treatment of AP-12 was investigated in wild type C57BL/6J mice and transgenic Alzheimer’s disease model mice (Tg APP_SweDI_) using behavioral tests and immunohistochemistry, as well as mass spectrometry to assess the blood-brain barrier (BBB) penetration. The data demonstrated the ability of AP-12 to cross the BBB, improve spatial learning and memory in both mice strains, induce anxiolytic action in transgenic mice, and increase expression of hippocampal and cortical proteins (GAD67, Homer-1) related to synaptic plasticity. The compound AP-12 can be seen as a prototype molecule for use in the design of novel drugs useful to halt progression of clinical symptoms (more specifically, anxiety and decline in memory) of neurodegenerative diseases, particularly Alzheimer’s disease.

## Introduction

Substantial evidence indicates that neuronal calcium signaling is abnormal in the pathogenesis of neurodegenerative disorders, such as Alzheimer’s disease, and dysregulated intracellular Ca^2+^ may cause a decline in cognitive functions [1–3]. Hence, Ca^2+^ blockers are suggested as beneficial not only in the traditional treatment of cardiovascular system pathologies [4] but also in neurology to treat neurodegenerative disorders [2,3,5]. Among Ca^2+-^channel blocking drugs, 1,4-dihydropyridine derivatives (DHP) have been shown to be able to penetrate the blood–brain barrier (BBB), and to reduce the risk of developing Parkinson’s [6–8] and Alzheimer’s disease [9].

**Fig 1:**
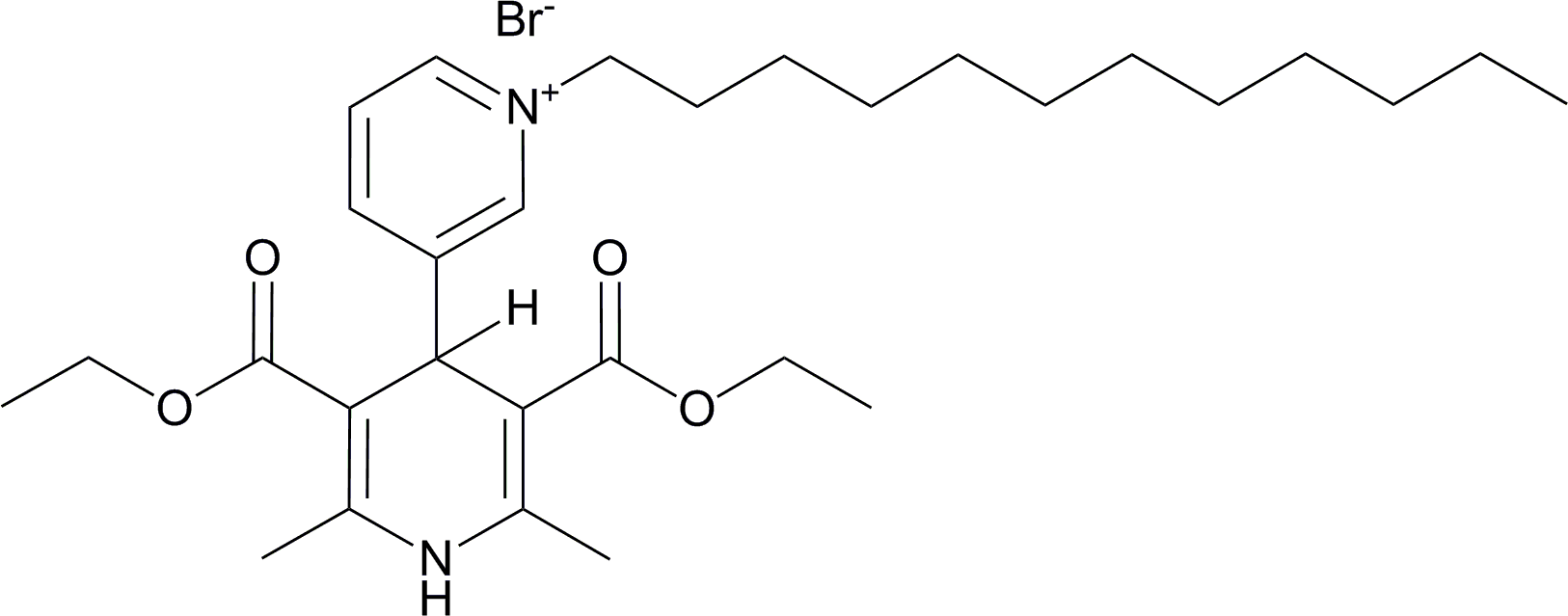
The chemical structure of AP-12.

The present study used the novel 4-(N-dodecyl) pyridinium group-containing DHP compound AP-12 (Fig 1). The rationale behind our research was to investigate AP-12 because it has previously been demonstrated that this compound has calcium antagonistic properties in neuroblastoma SH-SY5Y cells (IC50 = 5 ± 0.8 μM) and in the vascular smooth muscle A7r5 cell line (IC50 = 0.7 ± 0.3 μM) [10] that ranks this compound among calcium regulating DHPs. Furthermore, structural properties of this compound seem to be essential for the improvement of penetration through membranes to facilitate delivery to cellular targets. These properties are the dodecyl substituent as the lipophilic moiety, which was found as optimal length for gene transfection agents based on 1,4-DHP derivatives [11] and the DHP ring as the carrier molecule [12–14]. Previously it was shown that some DHP compounds may penetrate cell membranes, even those of organelles, and reach mitochondria and nucleus [15]. Therefore we hypothesized that the compound AP-12 would be able to modify protein expression in brain cells and hence, may influence cognitive functions. Up to now, the effects of DHPs on brain protein expression have not been studied, with exception to change calcium channel protein expression in the brain [16] and to protect against azidothymidine-induced overexpression of NF-kBp65 and caspase-3 in mouse brain [17]. It has been shown that neuronal calcium channels play an important role in learning and memory [18].

The present study investigated the ability of AP-12: 1) to improve cognitive functions in wild type C57BL/6J mice and Alzheimer’s disease model Tg APP_SweDI_ mice; 2) to penetrate the BBB in C57BL/6J mice, 3) to affect amyloid-beta deposition in transgenic mice; 4) to influence the expression of hippocampal and cortical proteins: Homer-1, GAD67 (glutamate decarboxylase 67), and AChE (acetylcholinesterase). These biomarkers were chosen because they play an important role in strengthening synaptic connections, that are considered as fundamental for the formation and maintenance of memory [19]. Homer-1 belongs to the post-synaptic density protein family, which is involved in neuronal synaptic transmission by mediating postsynaptic signalling transduction [20,21]. For instance, Homer-1 interacts with metabotropic glutamate receptor mGluR5 which is implicated in synaptic function and plasticity [21,22]. GAD67 is the enzyme that catalyzes the decarboxylation of glutamate to GABA. It has been shown that increasing GABA protects from neural injury [23], possibly by reducing overexcitability [24]. Moreover, GABAergic neurons are known to contribute to memory stabilization and consolidation of memory [25,26]. AChE, an acetylcholine degrading enzyme, was assessed as the protein controlling cholinergic activity that is known to play a role in hippocampal plasticity and memory formation [27]. Amyloid-beta (Aβ) extracellular accumulation in neuritic plaques is considered a neuropathological marker for Alzheimer's disease [28].

The data demonstrate that AP-12 was able to penetrate the BBB, improved learning and spatial memory in wild and transgenic mice, as well as induced anxiolytic action in transgenic mice. AP-12 modified GAD67 expression in both mice strains but did not alter Aβ deposits.

## Materials and Methods

### Animals and housing

For this study we used male C57BL/6J mice weighing 20-23g (n = 30) and male transgenic Alzheimer’s disease APP_SweDI_ mice (Tg APP_SweDI_) [29] weighing 25-30g (n = 16), both at 3 months of age. C57BL/6J mice were obtained from The Jackson Laboratory, Bar Harbor, ME, USA and Tg APP_SweDI_ mice were obtained from the breeding colony at the University of Alabama at Birmingham, USA. The animals were housed 5 per cage in a controlled environment at the University of Alabama at Birmingham Animal Care Facility (temperature 22°C, humidity 50–60%, and with a 12-h light/dark cycle) till the beginning of the experiment, and received food and water *ad libitum*. All efforts were made to minimize animal suffering and to reduce the number of animals used. Animal protocol for this study was approved by the University of Alabama at Birmingham Institutional Animal Care and Use Committee (IACUC), and approved protocol was strictly adhered to.

### Drugs and experimental design

The 1,4-dihydropyridine derivative AP-12 (3’,5’-bisethoxycarbonyl-2’,6’-dimethyl-1-dodecyl-1’,4-dihydro[3,4’]bipyridinyl-1-ium bromide), MW 579.61, was synthesized at the Latvian Institute of Organic Synthesis, Riga, Latvia [10]. For injections, AP-12 was dissolved in saline (with 0.1% Tween and using ultrasonication). AP-12 has a purity of 98% according to high performance liquid chromatography. The doses of 0.1 and 1 mg/kg of AP-12 were chosen as those demonstrating memory-enhancing effects obtained by studies of other DHP derivatives [30–32].

**Fig 2:**
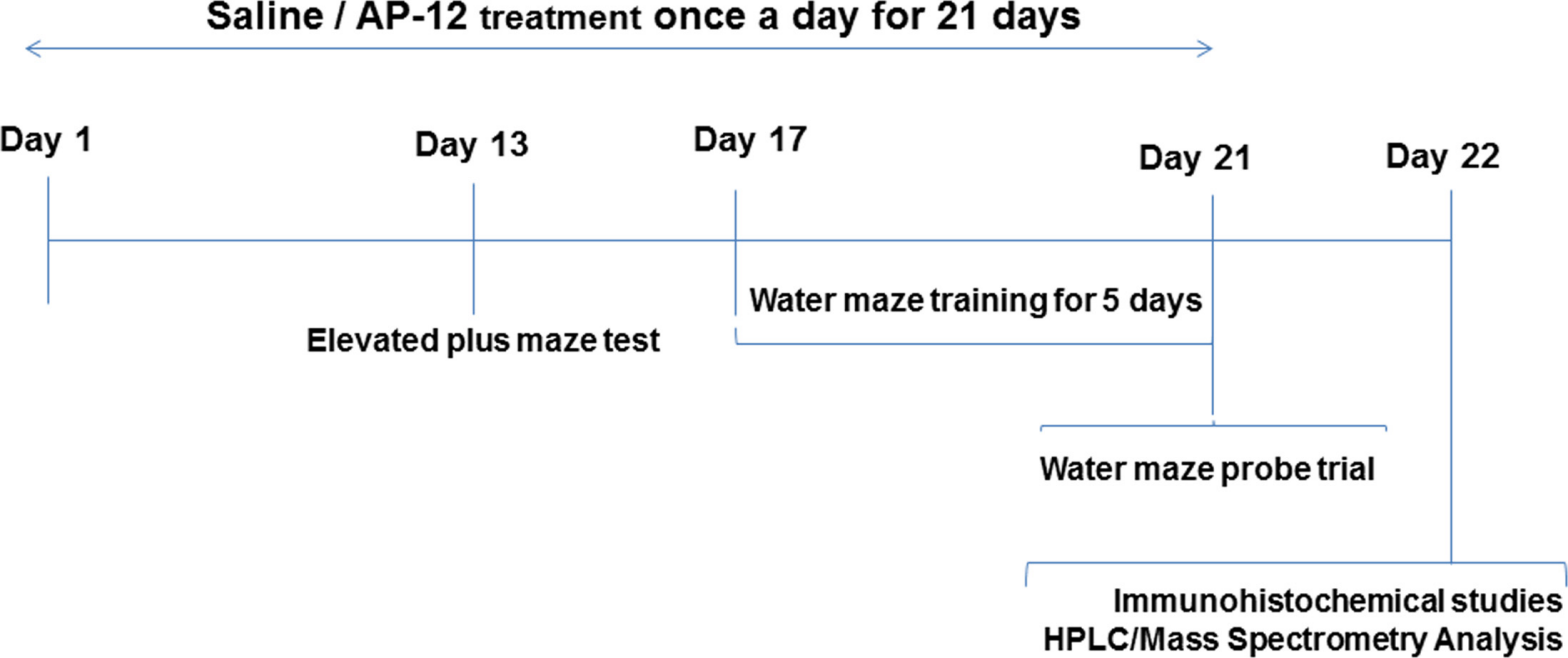
Experimental design.

Two separate studies were carried out, one on C57BL/6J mice and other on Tg APP_SweDI_ mice. The experimental design (Fig 2) was similar for both studies.

In the first study, male C57BL/6J mice were randomly divided into 3 groups (n = 10 per group): 1) control (saline with 0.1% Tween), 2) AP-12, 0.1mg/kg, and 3) AP-12, 1mg/kg. In the second study, male Tg APP_SweDI_ mice were randomly divided into 2 groups (n = 8 per group): 1) control (saline) and 2) AP-12, 1mg/kg. Saline or AP-12 were administered intraperitoneally (ip) once daily for 21 days. Thirteen days after the start of the treatment the animals were tested in elevated plus maze. From day 17 of AP-12 treatment, water maze test was performed for 5 days. All behavioral tests were done 3 h after AP-12 or saline injection and conducted between 11:00 and 14:00. At the end of the behavioral testing, i.e., on day 22 after the start of the treatment, mice were sacrificed; they were deeply anesthetized with ketamine/xylazine and perfused transcardially with cold saline for immunohistochemical and HPLC/Mass spectrometry analysis. Brains were removed immediately after perfusion. For immunohistochemical studies brains were immersed in 4% buffered paraformaldehyde for 24 h, cryoprotected in 30% sucrose in 0.1M phosphate buffer and stored in antifreeze solution at -20°C until the time of sectioning. For HPLC/Mass spectrometry analysis, brains from mice treated for 21 days with AP-12 were frozen and kept at -80°C until the assessment.

### Behavioral tests for C57BL/6J and Tg APP_SweDI_ mice

To track the activity and movements of the animals in the elevated plus maze and water maze a camera driven tracker system, i.e., Ethovision 8.5 (Noldus, The Netherlands) was used.

#### Elevated plus maze test

Briefly, the elevated plus maze consists of two open arms (31 cm×5 cm) and two closed arms (31 cm× 5 cm×15 cm) extended from a central platform (5 cm×5 cm), and was raised 40 cm above the table. On day 13 after the start of AP-12 treatment, the experiment was started by placing the mouse onto the central platform of the plus maze facing one of the open arms. The mouse was allowed to explore the maze for 4 min and then returned to its home cage. Distance and the time spent in the closed and open arms, and the total number of arm entries was recorded. An increase in time on the open arms and the number of entries to the open arms indicate anxiolytic action of the drug.

#### Water maze test

Our version of the water maze consisted of a blue circular tank (diameter 120 cm, height 40 cm) filled with clear water (23 ± 1°C). In the water maze test, mice were trained for four 60 s trials per day for 5 days to swim from one of the four starting points of the pool to find a fixed, hidden platform submerged below the water (0.5 cm) in one of the four quadrants of the pool. If the animal did not locate the platform during that time, it was placed upon platform by the experimenter and left there for 10 s. Inter-trial time was 1 min. The latency to find the hidden platform (escape latency) was recorded for each trial; similarly distance and speed were analyzed for each trial.

In the probe trial, which was performed after the last trial on day 5, the platform was removed from the pool and animals were allowed to swim for 60 s. The time spent in each quadrant was determined; similarly distance and speed were analyzed.

### Immunohistochemistry

The brain was sectioned coronally (6 series) at 30 μm on a freezing sliding microtome. Free-floating sections were then stained with the following primary antibodies (all of them from Millipore, Temecula, CA, USA): W0-2 antibody (mouse anti-human Aβ_4–10_; 1:5,000), Homer-1 (rabbit anti Homer1, 1:500, ABN37) and GAD67 (mouse anti-GAD67, 1:1000; MAB5406). In short, a series of sections was transferred to a solution containing the primary antibody; this solution consists of TBS pH 7.4 with 0.5% Triton X-100 added (TBS-T). Following incubation in this solution for 18 h on a shaker table at room temperature (20°C), the sections were rinsed three times in TBS-T and transferred to the solution containing the appropriate secondary antibody (goat anti-mouse*biotin; Sigma or goat anti-rabbit*biotin; Millipore, Temecula, CA, USA). After 2 h, the sections were rinsed three times with TBS-T and transferred to a solution containing mouse ExtrAvidin (1:1000; Sigma, Saint Louis, MO, U.S.A.) for 2 h. Following rinsing, the sections were incubated for 3 min with Ni-enhanced DAB. All stained sections were mounted on gelatin-coated slides and coverslipped. It should be noted that to obtain similarly stained material, sections from all animal groups were always stained simultaneously in the same staining tray.

One series of sections was stained for AChE according to a modified method of Koelle and Friedenwald [33,34], mounted and coverslipped.

### Quantification

The appropriate areas (dorsal hippocampus; Bregma– 2.18 and prefrontal cortex; Bregma + 0.50, according to Franklin and Paxinos, 1997) of the brains were digitized using an Olympus DP70 digital camera. To avoid changes in lighting, which might affect measurements, all images were acquired in one session. Furthermore, to avoid differences in staining density between sections, the measurements were performed on sections that were stained simultaneously, i.e., in the same staining tray. The percentage of area covered by the reaction product to Aβ was measured [35] in hippocampus (stratum oriens of CA1 and the dentate gyrus); the density of Homer-1 and GAD67 staining was measured by the optical density of the staining in the appropriate hippocampal and prefrontal cortex areas using the ScionImage (NIH) program [36]. The density of AChE staining was measured by the optical density in the appropriate hippocampal area (stratum pyramidale and radiatum of area CA1). All density measurements were done in duplicate. These measurements were done by an observer blinded to the treatment of the animal.

### Brain samples preparation for quantitative analysis by mass spectrometry

For the quantitative analysis by mass spectrometry (LCMS-MRM) C57BL/6J mouse brains of the treatment group receiving AP-12 at the dose of 1mg/kg were used. Sample homogenization of whole mice brain was performed using a mechanical homogenizer followed by 10 min of intermittent sonication in an ice bath. For the extraction of AP-12, the homogenized brain tissue (0.2 ml) was placed into a 13x100 mm glass tube and extracted with 1.2 ml of chloroform. All tubes were capped, vortexed twice, and placed onto a rotating shaker at 37° for 2 h. Each tube was removed from the rotating shaker and centrifuged at 3200 rpm for 5 minutes at 4°C (Jouan CR3 bench-top centrifuge.) After removal from the aqueous layer the chloroform layer had an approximate volume of 1 ml. The chloroform layer was transferred to a fresh glass tube and evaporated under nitrogen gas. The dried samples were reconstituted in 0.2 ml of 50% water, 50% acetonitrile, 0.1% formic acid. To determine the extraction yield of AP-12, brain tissue samples from an untreated mouse were spiked with AP-12 (0.1μg/ml) before and after extraction process.

### HPLC/Mass spectrometry analysis

An Applied Biosystems/Sciex (Toronto, Canada) API 4000 Q-TRAP triple-quadrupole mass spectrometer was used for this study. The Turbo Ion source was operated under positive electro spray at 650°C. All gases were nitrogen and the declustering potential (DP), collision exit potential (CXP) and collision energy (CE) were optimized for AP-12. Quantitative analysis was undertaken using a Shimadzu Class VP HPLC system. Liquid chromatography was performed using a Phenomenex (Torrance, CA, USA) Luna Phenyl-Hexyl 3 micron 2 x 30 mm HPLC column at 50°C. The injection volume was 5 μl and the flow rate was 1.0 ml/min. Solvent A was 0.1% formic acid in water and solvent B was acetonitrile with 0.1% formic acid. The total length of the gradient was 10 minutes. The gradient was held at the initial condition of 40% for one minute, and then had a linear increase to 100% B at 5 minutes. The gradient was then held at 100% B for an additional minute and returned to the initial condition at 6.1 minutes. The gradient was then held at the initial condition (40% B) until 10 minutes to equilibrate the column for the next injection.

### Statistical analysis

Behavioral data are expressed as mean ± SEM, and statistics were performed with GraphPad Prism 5 software (GraphPadSoftware Inc., CA, USA). For the statistical analysis of the data obtained in C57BL/6J mice study and water-maze data in Tg APP_SweDI_ mice, ANOVA followed by Bonferroni Multiple Comparison post-test was used. Elevated plus maze test data in Tg APP_SweDI_ mice study was analyzed with unpaired t test. In all cases, a *p*-value < 0.05 was taken as the criterion of statistically significant difference.

Immunohistochemical quantification data were analyzed by ANOVA (GraphPad Prism; between groups), and Bonferroni post-test were carried out to determine the source of a significant main effect or interaction. Statistical significance was set at *p*< 0.05.

## Results

### AP-12 improves spatial learning and memory in C57BL/6J mice

**Fig 3:**
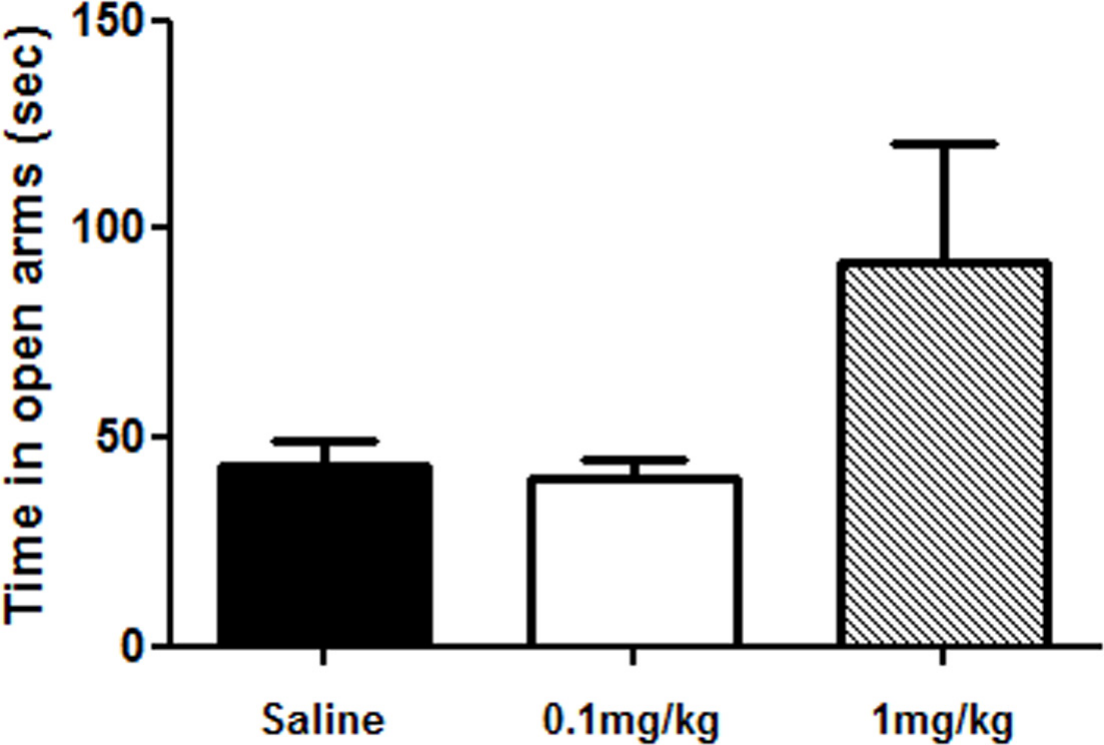
Time spent in open arms of the elevated plus maze of the AP-12-treated C57BL/6J mice (n = 10 mice/group). AP-12 was injected at doses 0.1 and 1 mg/kg ip once a day for 13 days. Elevated plus maze test was performed 3h after AP-12 or saline injections on day 13. Data represent time in seconds spent in the open arms ± SEM. No statistical significance was found between treatment and saline groups.

**Fig 4:**
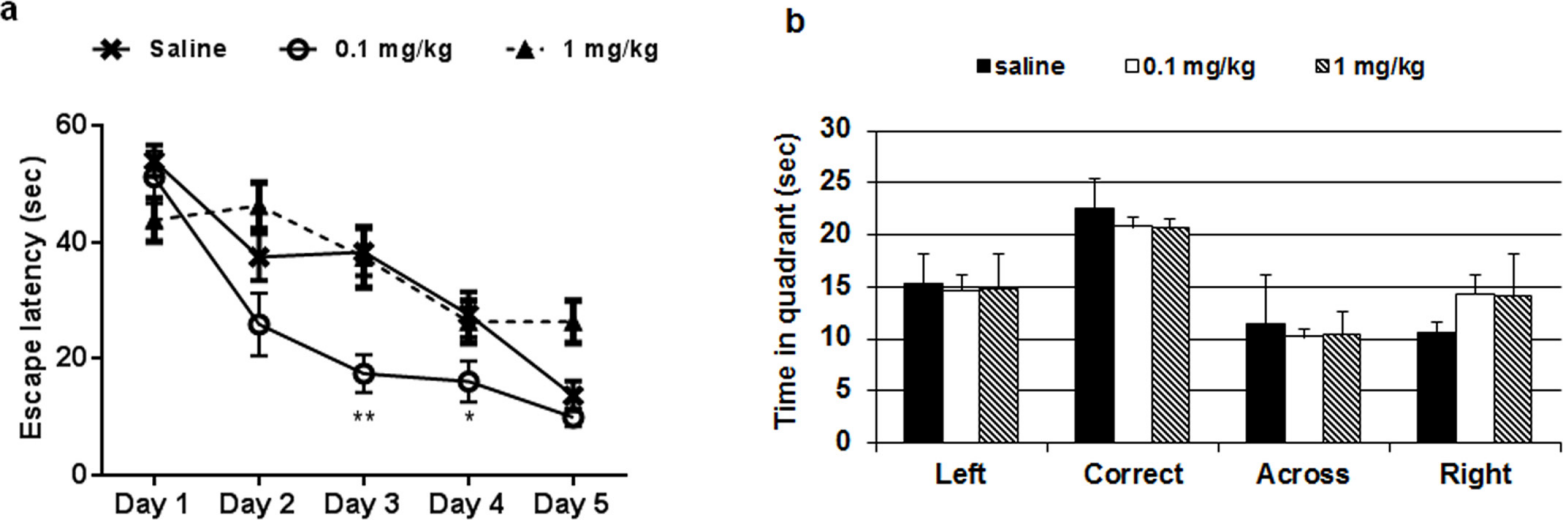
Effects of AP-12 (0.1 and 1 mg/kg) on spatial memory in C57BL/6J mice (n = 10 mice/group) in water maze test. The compound was injected once a day for 17 days. Water maze test was started 3h after AP-12 or saline injections on day 17. (A) Three line graphs showing the learning curves of the three groups of mice. Daily mean latency was calculated from four trials; values are mean ± SEM. Significance was measured by two-way ANOVA followed by Bonferroni Multiple Comparison post-test. *p<0.05 vs. saline control group, **p<0.01 vs. saline control group. (B) The probe trial test.

In the elevated plus maze test, administration of AP-12 (0.1 and 1 mg/kg; ip once daily) or saline for 13 days in C57BL/6J mice showed no significant difference in time spent in open arms (p = 0.08) (Fig 3, Table A in S1 File) and number of entries among groups. On day 17^th^ of treatment, the water maze test was started. AP-12 at the dose of 0.1 mg/kg significantly shortened platform latency vs. saline group on days 3 (p = 0.009) and 4 (p = 0.04) (Fig 4A); on day 2 only a tendency for learning faster was observed; similarly on day 5, no significant difference was present (Table B in S1 File).

In the probe trial, no difference in the preference for the target quadrant was present between the groups (Fig 4B, Table C in S1 File). No significant differences were observed in the swimming speed of treated mice vs saline control group data on day 4 (saline: 15.3 ± 1.4 cm/sec; AP-12, 0.1 mg/kg: 17.8 ± 1.9 cm/sec; AP-12, 1mg/kg: 13.9 ± 1.2 cm/sec) neither on any other days (Table D in S1 File).

### AP-12 upregulates GAD67 and Homer-1 expression in the brain of C57BL/6J mice

**Fig 5:**
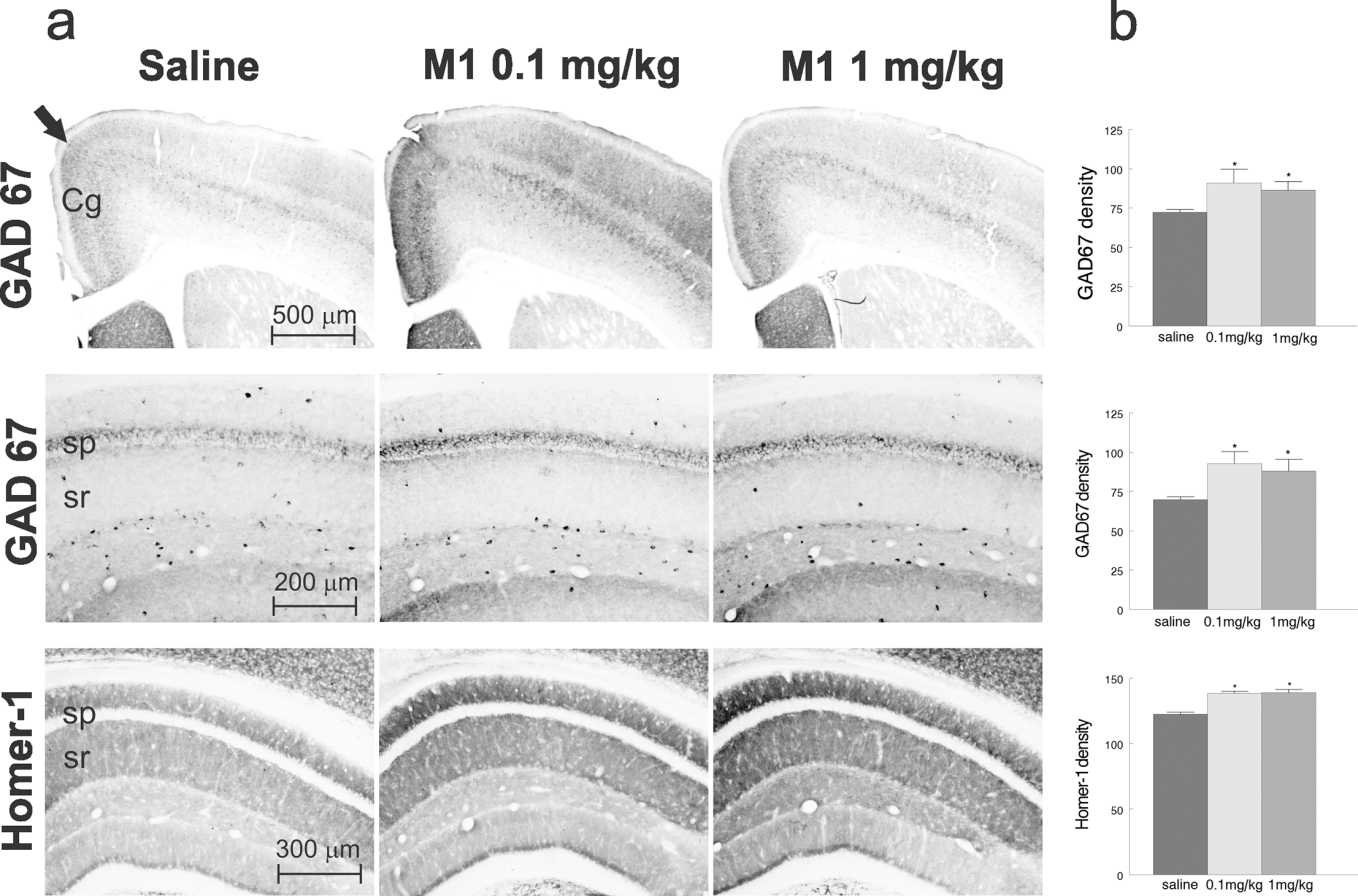
Photomicrographs of the cortex and hippocampus of C57BL/6J mice after treatment of saline (control), AP-12, 0.1 and 1 mg/kg. (A) Top row: three photomicrographs of coronal sections of the rostral, midline cortex (anterior cingulate cortex) stained for GAD67. The GAD67 staining density was significantly increased (p <0.05) in the cingulate cortex in both treatment groups compared to saline group. Middle row: three photomicrographs of the dorsal hippocampus stained for GAD67. The GAD67 staining density was significantly (p<0.05) increased in and around CA1 pyramidal cell layer in both treatment groups compared to saline group. The Homer-1 staining density was significantly increased (p <0.05) in stratum radiatum and stratum oriens in both treatment groups compared to saline group. Arrow in the top row indicates the border of cingulate cortex, Cg–cingulate cortex, sp–stratum pyramidale, sr–stratum radiatum. (B) Bar graphs showing the quantification of the density measurements.

Immunohistochemical analysis demonstrated that AP-12 treatment at doses 0.1 and 1 mg/kg altered Homer-1 and GAD67 expression in the hippocampus and cerebral cortex. The staining density for GAD67 was significantly increased around the CA1 pyramidal layer in the dorsal hippocampus (Fig 5A) in both AP-12 treatment groups (0.1 and 1 mg/kg) compared with the saline group (92.8 ± 7.5 and 88 ± 7.4, respectively vs. 69.8 ± 1.7; p< 0.05). GAD67 expression was also increased in the anterior cingulate cortex of 0.1 and 1 mg/kg treated mice (Fig 5A) compared with saline treated mice (91.0 ± 8.6 and 86.3 ± 5.4, respectively vs. 72.2 ± 1.4; p< 0.05). Similarly, the results of the Homer-1 stained brain sections showed a significant increase in the staining density in stratum radiatum and stratum oriens of area CA1 of the dorsal hippocampus (Fig 5A) in both 0.1 and 1 mg/kg treatment groups compared to the saline group (138.3 ± 1.6 and 139.1 ± 2.3, respectively vs. 122.5 ± 1.5; p< 0.05). Fig 5B represents bar graphs showing the quantification of the density measurements for the analyzed regions. There were no significant changes in the AChE staining density in these brain areas.

### AP-12 crosses the BBB in C57BL/6J mice

**Fig 6:**
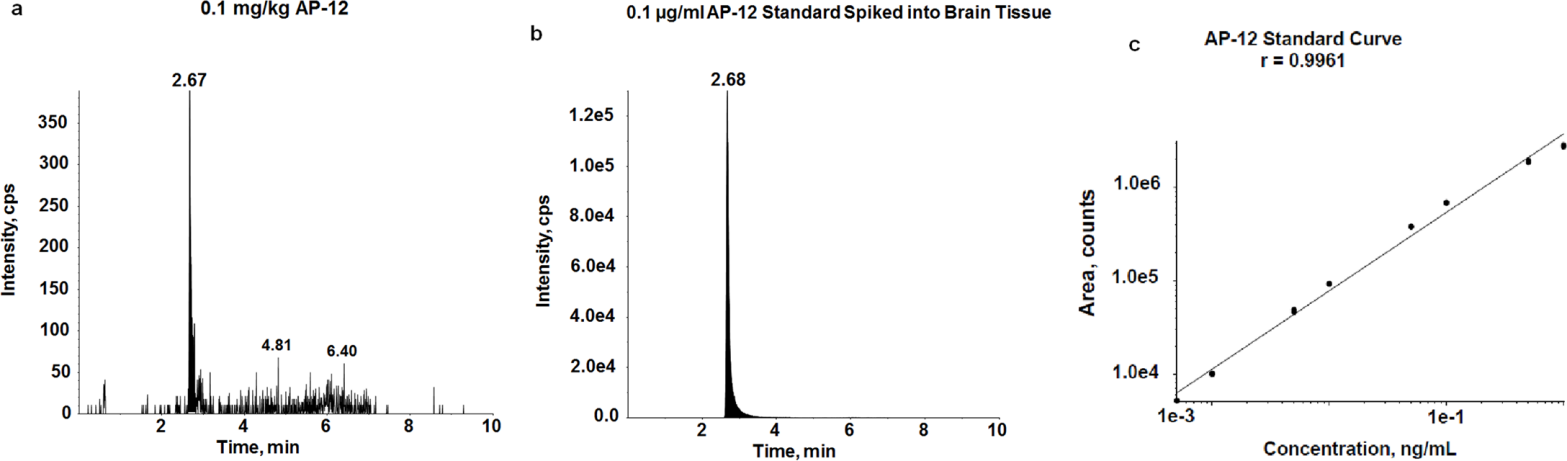
Blood-brain barrier permeability of AP-12 at the dose 1 mg/kg in C57BL/6J mice. (A) AP-12 detected in the whole brain homogenate by mass spectrometry (LCMS-MRM) analysis with the peak corresponding to AP-12 eluted from the column at 2.68 min. (B) AP-12 spiked into untreated mouse brain homogenate at 0.1 μg/ml and extracted. (C) AP-12 standard curve from 1.0 to 100 ng/ml (r = 0.9989).

The results of the mass spectrometry analysis demonstrated that AP-12 can cross the BBB. The chromatogram in Fig 6A shows that AP-12 was detected in whole brain homogenate from AP-12-treated mice. The peak corresponding to AP-12 eluted from the column at 2.68 min (Fig 6A) and the calculated concentration of AP-12 detected in the brain homogenates from a dose of 1mg/kg was 0.084 ng/ml. For these experiments, the mass transition used to quantify was 501.6 > 80.1. The pure standard of AP-12 as shown in Fig 6B was at the same retention time as the peak in Fig 6B. An endogenous peak was revealed at the same retention time in unspiked samples that was equal to approximately 18% of the measured peak in AP-12-spiked homogenates. A representative standard curve from 1.0 to 100 ng/ml (r = 0.9989) is shown in Fig 6C.

### Anxiolytic and learning and memory-improving activity of AP-12 in Tg APP_SweDI_ mice

**Fig 7:**
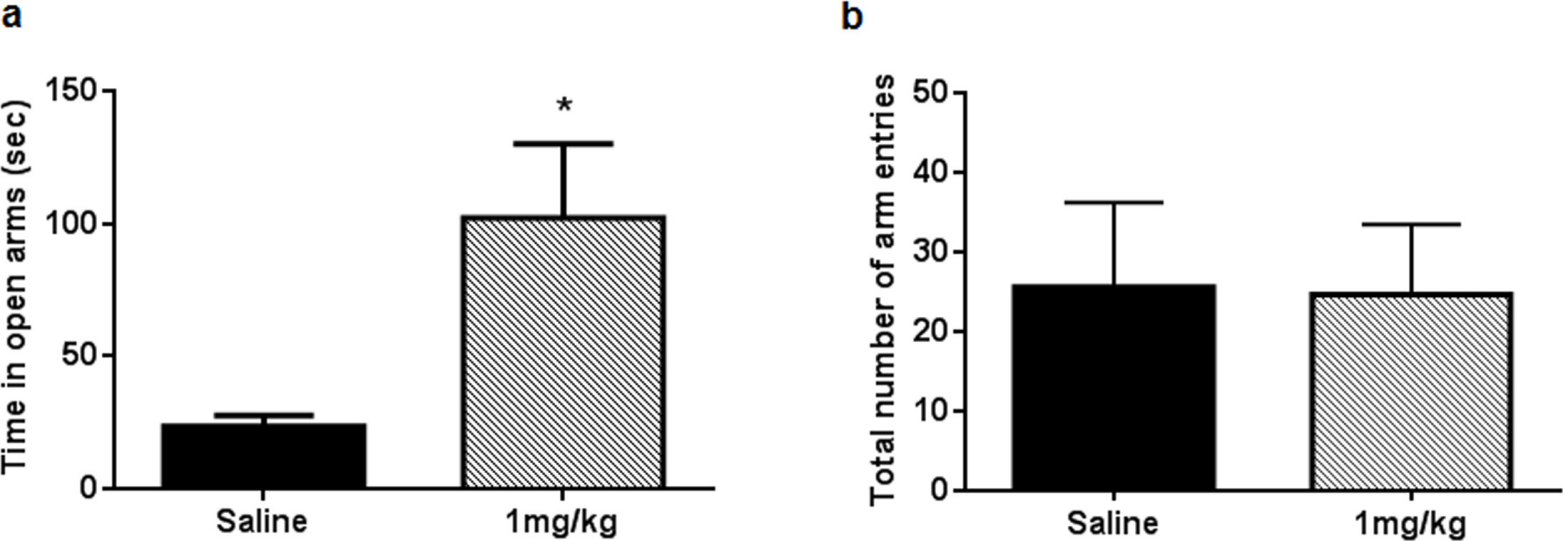
Time spent in open arms of AP-12-treated Tg APP_SweDI_ mice (n = 8 mice/group) in the elevated plus maze. AP-12 was injected at dose 1 mg/kg ip once a day for 13 days. Elevated plus maze test was performed 3 h after AP-12 or saline injection on day 13. (A) The data are expressed as time in seconds spent in the open arms. (B) The data represent total number of arm entries. Values are mean ± SEM. Significance was measured using unpaired t test. *p<0.05 vs. saline control group.

**Fig 8:**
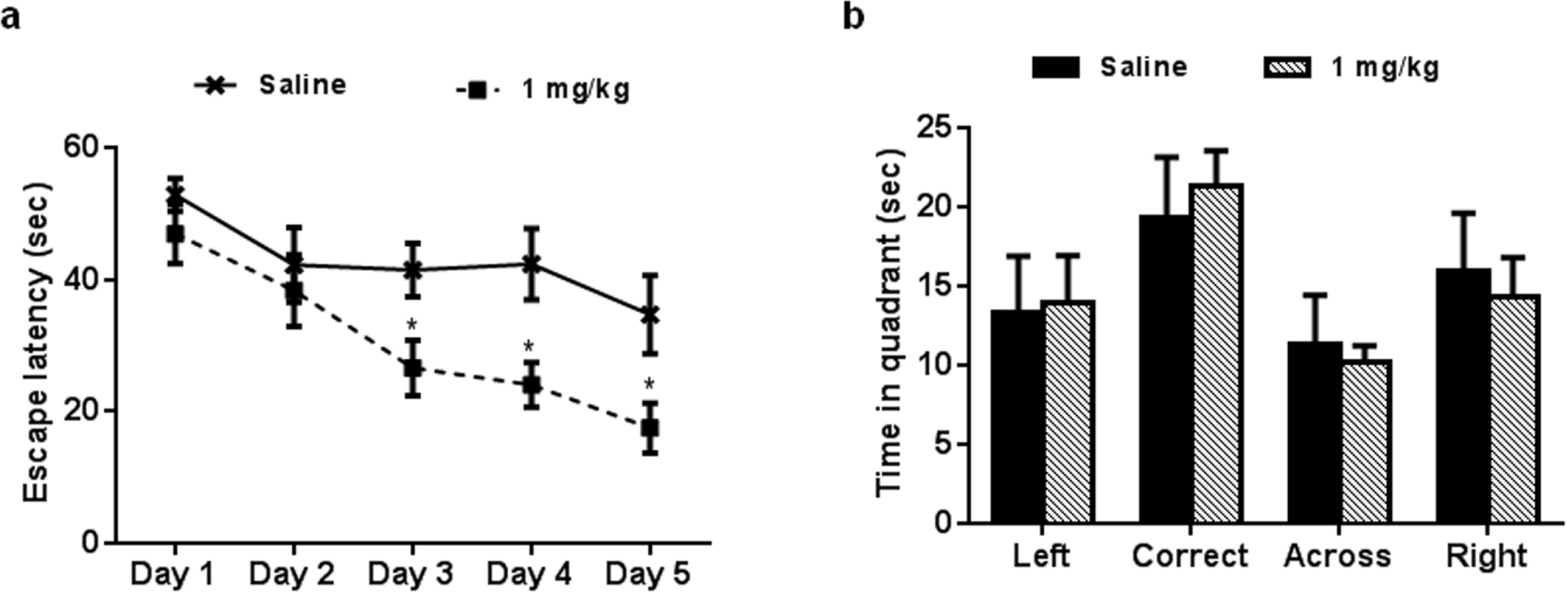
Effects of AP-12 (1mg/kg) on spatial memory in Tg APP_SweDI_ mice (n = 8 mice/group) in water maze test. The compound was injected once a day for 17 days. (A) Two line graphs showing the learning curves of the two groups of mice. Daily mean latency calculated from four trials. Values are mean ± SEM. Significance was measured by two-way ANOVA followed by Bonferroni Multiple Comparison post-test. *p<0.05 vs. saline control group. (B) The probe trial test.

The data showed that administration of AP-12 at the dose of 1 mg/kg (ip) significantly increased the time spent in open arms of the elevated plus maze compared to saline control group mice (p = 0.023) (Fig 7A, Table E in S1 File) whereas no significant difference (p = 0.84) in number of total arms entries (Fig 7B, Table F in S1 File) and in mean distance moved (saline: 1177.0 ± 98.07 cm; AP-12, 1 mg/kg: 1284,4 ± 85.5 cm; p = 0.49; Table G in S1 File) in arms were observed between groups. AP-12 treatment improved the spatial learning performance in water maze test by shortening the latency to find the hidden platform (vs. saline control) on days 3–5 (p = 0.037; p = 0.023 and p = 0.044, respectively) (Fig 8A, Table H in S1 File). In the probe trial no difference in the preference for the target quadrant was present between the groups (Fig 8B, Table I in S1 File). There were no significant differences in the swimming speed of the mice between the two groups on day 4 (saline: 12.7 ± 1.8 cm/sec and AP-12: 14.2 ± 0.9 cm/sec) and all other days either (Table J in S1 File).

### AP-12 upregulates GAD67 expression in brain of Tg APP_SweDI_ mice

**Fig 9:**
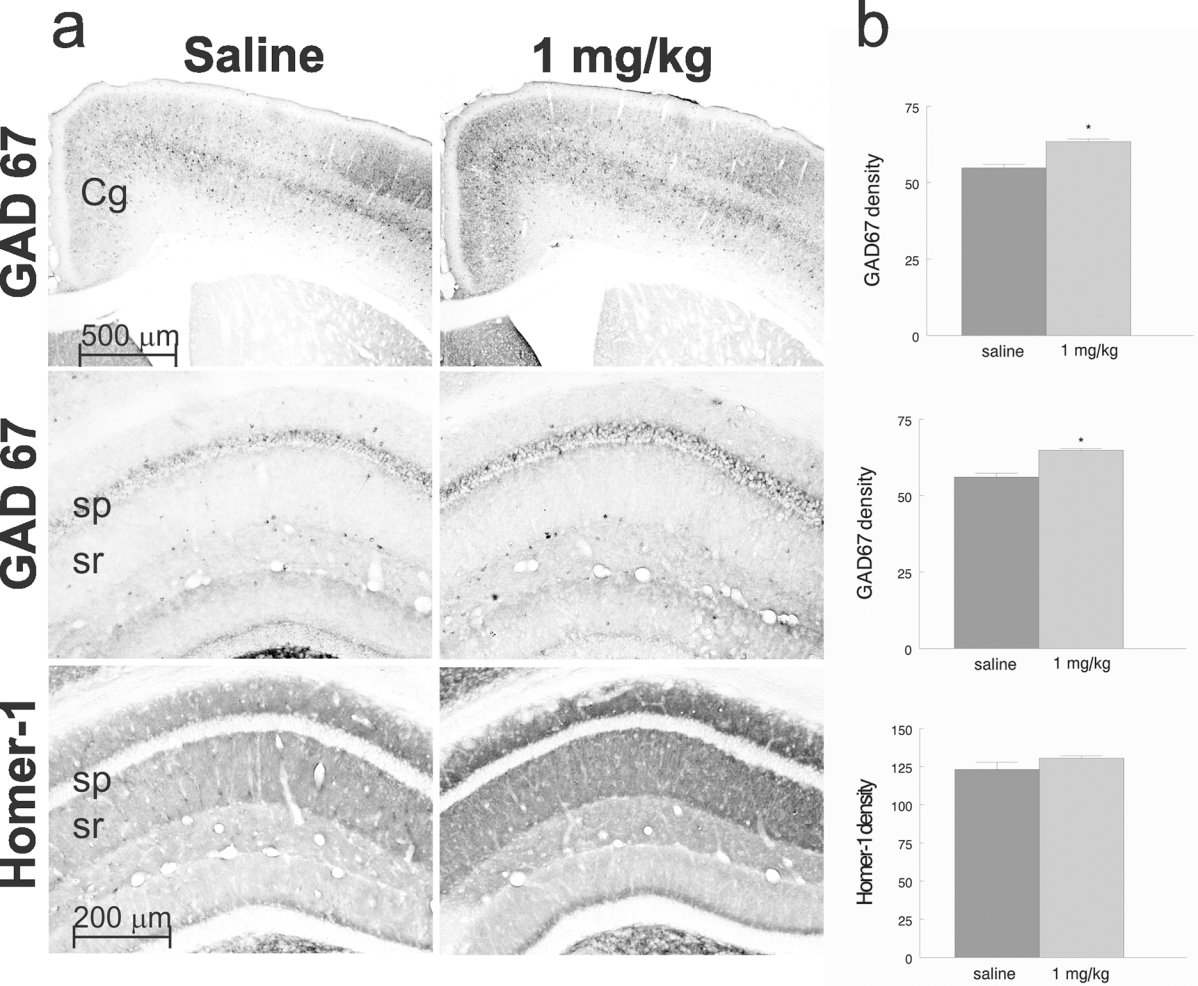
Photomicrographs of the cingulated cortex and dorsal hippocampus of Tg APP_SweDI_ mice after treatment with saline (control) or AP-12 (1 mg/kg). (A) Top row: two photomicrographs of coronal sections of the rostral, midline cortex (anterior cingulate cortex) stained for GAD67. The GAD67 staining density was significantly increased (p<0.05) in cingulate cortex in AP-12 group compared to saline group. Middle row: two photomicrographs of the dorsal hippocampus stained for GAD67. The GAD67 staining density was significantly (p<0.05) increased around CA1 pyramidal cell layer in the treatment group compared to saline group. Bottom row: two photomicrographs of the dorsal hippocampus stained for Homer-1. The Homer-1 staining density was not significantly different between AP-12 and saline groups. Cg–cingulate cortex, sp–stratum pyramidale, sr–stratum radiatum. (B) Bar graphs showing the quantification of the density measurements.

Immunohistochemical staining (Fig 9A and 9B) showed that AP-12 at the dose of 1 mg/kg significantly increased GAD67 immunoreactivity density in the cingulate cortex (64.9 ± 0.6 vs. 56.0 ± 1.3; p< 0.05) and hippocampus (63.5 ± 0.9 vs. 54.9 ± 1.1; p<0.05) compared to saline injected animals. However, AP-12 administration did not significantly change Homer-1 expression. There were no changes in the amyloid beta load and AChE staining density in hippocampus and cingulate cortex of the treated mice compared with saline treated-animals (data not shown).

## Discussion

Previously we have shown a wide spectrum of effects of some DHP compounds tested at doses 0.05–1 mg/kg, ip: memory-enhancing [31,37], anti-neuroinflammatory [17,38], and mitochondria-protection [39]. These different effects of DHP compounds are likely related to its interactions with diverse receptors and ion channels [40]. Because of the ability of DHP derivates to reach the nucleus [40] we suggest that compounds of this series may interfere with intracellular machinery regulating brain protein expression.

In this context, we studied whether AP-12, the DHP compound under study, may penetrate the BBB, whether and how it may influence brain protein expression, and does this correlates with cognitive responses.

The results from the mass spectrometry analysis of the brains of C57BL/6J mice showed, that AP-12 (1 mg/kg) can cross the BBB, and was detectable as 0.084 ng/ml in the brain homogenate. One may consider that the structure and physicochemical properties of AP-12 –lipophilic 4-(N-dodecyl) pyridinium group and the scaffold of DHP ring as carrier moiety–were essential to provide its penetration through the BBB. The amount that is present in the brain is enough to induce alterations in brain protein expression and the enhancement of learning and memory. The BBB-crossing ability of DHP derivates is considered to be crucial for drugs of this series with memory-improving action in Parkinson’s disease [6–8] and Alzheimer’s disease [9].

In the first study, with C57BL/6J mice, AP-12 at the dose 1 mg/kg but not at the dose of 0.01 mg/kg showed a tendency to have an anxiolytic effect (0.08 vs control group) in the elevated plus maze test. Further, chronic administration of AP-12 at dose 0.1 mg/kg but not at dose 1 mg/kg) significantly improved spatial learning and memory in water-maze test on training days 3 and 4. On day 5 no significant difference was seen, likely because optimal performance was reached in both groups. No significant differences in the probe trial were observed, probably because a significant number of the mice uses a search strategy (instead of knowing where the platform is) to find the escape platform. Further, both doses caused changes in the expression of hippocampal GAD67 and Homer-1 proteins, indicating that protein expression is more sensitive and may appear even if the changes in behavior are not seen. Along these lines, a recent report demonstrated changes in the BDNF expression prior to onset of motor behavior abnormalities in Huntington’s disease model mice [41].

For the studies in Tg APPSweDI mice the higher dose (1 mg/kg) was chosen, since we assumed that cognition and pathology would not be changed by the lower dose of AP-12. In this study, chronic administration of AP-12 showed similar effects in comparison to those obtained in wild type mice. AP-12 had an anxiolytic effect in the elevated plus-maze test, it increased the time spent in open arms while mean distance moved was similar between groups. Further, it improved spatial learning and memory on training days 3–5 in water maze test. Similar to the C57BL\6J mice, no significant differences were seen in the probe trial. The lack of significant differences in the probe trial is probably caused by because a significant number of the mice uses a search strategy (instead of knowing where the platform is) to find the escape platform [42].

Immunohistochemical data showed that AP-12 upregulated GAD67 (but not Homer-1) expression in the cingulate cortex and hippocampus in the Tg AD model mice.

Hippocampus is considered as the brain structure playing a pivotal role in spatial learning and memory [43,44]. The increase in GAD67 expression suggests that AP-12 may promote GABA synthesis. In turn, GABAergic neurons are known to contribute to memory stabilization and consolidation of memory [25,26]. The role of GABAergic processes was shown previously for another memory-enhancing DHP compound, cerebrocrast [31]. The enhancement of GABAergic activation is important because reduced GABA transmission has been reported in human Alzheimer’s disease brain [45]. It is known that progressive decline in memory, anxiety and depression are very characteristic symptoms in Alzheimer’s disease patients [46] and the necessity to reduce them together with memory improvement [47] is indisputable. Hence both properties of the compound AP-12 –anxiolytic and memory-improving–are a good combination to rescue impairments in Alzheimer’s disease.

In contrast to the changes in GAD67 and Homer1 expression, treatment with AP-12 did not induce significant changes in AChE expression neither in wild type nor in transgenic mice. AP-12 also did not change the amyloid beta (Aβ) load in the brains of transgenic AD mice. The hippocampus is a brain area that develops early amyloid deposits in the course of AD [48]. The question which structures of DHP compounds can decrease Aβ burden is not clear. For instance, among DHPs, such as nilvadipine, amlodipine and nifedipine, all with antihypertensive activity and memory-improving abilities, only nilvadipine was found to reduce Aβ levels [49]. Finally, we cannot exclude cerebrovascular dilating activity of AP-12, as it showed calcium channel blocking activity in a vascular cell line [10].

In summary, the present data demonstrated that AP-12, a calcium antagonist of DHP series, can penetrate the BBB, it improves spatial learning and memory in wild type mice and Tg APP_SweDI_ model mice, and it has an anxiolytic effect in Tg APP_SweDI_ mice. We demonstrate that the DHP derivative changes the levels of brain proteins which contribute to synaptic plasticity, GAD67 and Homer1. Our data indicate that AP-12 treatment is able to alleviate some of the anxiety and learning and memory problems in Tg APP_SweDI_ model mice, however more studies are necessary to elucidate the molecular mechanisms through which the beneficial effects are achieved. In addition, AP-12 can be considered as a prototype molecule for the design of novel drugs capable to halt progression of clinical symptoms (more specifically anxiety and memory impairment) of neurodegenerative pathologies, particularly Alzheimer’s disease.

## Supporting Information

S1 File Contains Tables A-J. (DOCX)
