# Supplementary material for: A Novel 1,4-Dihydropyridine Derivative Improves Spatial Learning and Memory and Modifies Brain Protein Expression in Wild Type and Transgenic APPSweDI Mice
Source: PLoS One. 2015 Jun 4;10(6):e0127686. doi: 10.1371/journal.pone.0127686 (PMC4456351; doi:10.1371/journal.pone.0127686)
Supplement: S1 File — (DOCX) [file pone.0127686.s001.docx]

**Table A.** Time (sec) spent in open arms of the elevated plus maze of the AP-12-treated C57BL/6J mice. Values presented as mean ± SEM.

| **Saline** | **AP-12, 0.1 mg/kg** | **AP-12, 1 mg/kg** |
| --- | --- | --- |
| 43.53 **±** 5.89 | 40.54 **±** 4.45 | 49.01 **±** 9.46 |

**Table B.** Effects of AP-12 (0.1 and 1 mg/kg) on spatial memory in C57BL/6J mice in water maze test. Daily mean latency was calculated from four trials; values are mean ± SEM.

|  | **Day 1** | **Day 2** | **Day 3** | **Day 4** | **Day 5** |
| --- | --- | --- | --- | --- | --- |
| **Saline** | 53.94 ± 2.70 | 37.41 ± 4.00 | 38.28 ± 4.05 | 27.57 ± 3.88 | 13.62 ± 2.54 |
| **AP-12,**  **0.1 mg/kg** | 51.10 ± 4.50 | 25.88 ± 5.37 | 17.40 ± 3.23 | 16.05 ± 3.53 | 9.92 ± 1.58 |
| **AP-12,**  **1 mg/kg** | 43.76 ± 3.69 | 46.23 ± 3.98 | 37.39 ± 5.38 | 26.31 ± 3.32 | 26.51 ± 3.41 |

**Table C.** The probe trial test in C57BL/6J mice. Time in quadrant (sec), values presented as mean ± SEM.

|  | **Left** | **Correct** | **Across** | **Right** |
| --- | --- | --- | --- | --- |
| **Saline** | 15.34 ± 2.80 | 22.55 ± 2.80 | 11.41 ± 4.78 | 10.54 ± 1.14 |
| **AP-12,**  **0.1 mg/kg** | 14.61 ± 1.46 | 20.77 ± 0.98 | 10.16 ± 0.84 | 14.31 ± 1.82 |
| **AP-12,**  **1 mg/kg** | 14.77 ± 3.33 | 20.58 ± 1.01 | 10.45 ± 2.16 | 14.056 ± 4.04 |

**Table D** Swimming speed (cm/sec) of AP-12-treated C57BL/6J mice in water maze test represented by day 4 values. Values are mean ± SEM.

| **Saline** | **AP-12, 0.1 mg/kg** | **AP-12, 1 mg/kg** |
| --- | --- | --- |
| 15.3 **±** 1.4 | 17.8 **±** 1.9 | 13.9 **±** 1.2 |

**Table E.** Time (sec) spent in open arms of the elevated plus maze of the AP-12-treated Tg APP_SweDI_ mice. Values presented as mean ± SEM.

| **Saline** | **AP-12, 1 mg/kg** |
| --- | --- |
| 23.51 **±** 4.24 | 102.10 **±** 27.86 |

**Table F.** Total number of arm entries of AP-12-treated Tg APP_SweDI_ mice in the elevated plus maze. Values presented as mean ± SEM.

| **Saline** | **AP-12, 1 mg/kg** |
| --- | --- |
| 25.60 **±** 3.34 | 24.63 **±** 3.13 |

**Table G.** Mean distance moved of AP-12-treated Tg APP_SweDI_ mice in the elevated plus maze. Values presented as mean ± SEM.

| **Saline** | **AP-12, 1 mg/kg** |
| --- | --- |
| 1177.01 **±** 98.07 | 1284.41 **±** 85.52 |

**Table H.** Effects of AP-12 (1 mg/kg) on spatial memory in Tg APP_SweDI_ mice in water maze test. Daily mean latency was calculated from four trials; values are mean ± SEM.

|  | **Day 1** | **Day 2** | **Day 3** | **Day 4** | **Day 5** |
| --- | --- | --- | --- | --- | --- |
| **Saline** | 52.83 ± 2.45 | 42.20 ± 5.67 | 41.42 ± 4.06 | 42.34 ± 5.43 | 34.68 ± 5.94 |
| **AP-12,**  **1 mg/kg** | 46.91± 3.83 | 38.30 ± 4.66 | 26.59 ± 3.39 | 24.00 ± 2.91 | 17.46 ± 3.23 |

|  |  |
| --- | --- |

**Table I.**The probe trial test for Tg APP_SweDI_ mice. Time in quadrant (sec), values presented as mean ± SEM.

|  | **Left** | **Correct** | **Across** | **Right** |
| --- | --- | --- | --- | --- |
| **Saline** | 13.29 ± 3.58 | 19,30 ± 3.82 | 11.35 ± 3.07 | 15.90 ± 3.68 |
| **AP-12,**  **1 mg/kg** | 13.94 ± 2.96 | 21,35 ± 2.18 | 10.21 ± 1.01 | 14.34 ± 2.44 |

**Table J.** Swimming speed (cm/sec) of AP-12-treated Tg APP_SweDI_ mice in water maze test represented by day 4 values. Values are mean ± SEM.

| **Saline** | **AP-12, 1 mg/kg** |
| --- | --- |
| 12.7 **±** 1.8 | 14.2 **±** 0.9 |
